# Overexpressed integrin alpha 2 inhibits the activation of the transforming growth factor β pathway in pancreatic cancer via the TFCP2-SMAD2 axis

**DOI:** 10.1186/s13046-022-02286-5

**Published:** 2022-02-22

**Authors:** Hongkun Cai, Feng Guo, Shuang Wen, Xin Jin, Heshui Wu, Dianyun Ren

**Affiliations:** 1 Department of Pancreatic Surgery, Union Hospital, Tongji Medical College, Huazhong University of Science and Technology, Wuhan, 430022 China; 2 Sino-German Laboratory of Personalized Medicine for Pancreatic Cancer, Union Hospital, Tongji Medical College, Huazhong University of Science and Technology, Wuhan, 430022 China; 3 Department of Emergency Medicine, Union Hospital, Tongji Medical College, Huazhong University of Science and Technology, Wuhan, 430022 Hubei China; 4 Department of Urology, The Second Xiangya Hospital, Central South University, Changsha, 410011 Hunan China

**Keywords:** Pancreatic cancer, Integrin α2, Transcription factor CP2, SMAD2, TGF-β

## Abstract

**Background:**

Integrin alpha 2 (ITGA2) has been recently reported to be an oncogene and to play crucial roles in tumor cell proliferation, invasion, metastasis, and angiogenesis. Our previous study showed that ITGA2 was overexpressed in pancreatic cancer and promoted its progression. However, the mechanism of ITGA2 overexpression and other mechanisms for promoting the progression of pancreatic cancer are still unclear.

**Methods:**

The GEPIA database was used to confirm the expression of ITGA2 in pancreatic cancer. To verify the influence of ITGA2 and TGF-β on the morphological changes of pancreatic cancer and tumor cell progression, we conduct CCK8 test, plate cloning, flow cytometry experiments and animal experiments. Then we conduct Western blot, RT-qPCR to explore the relationship between ITGA2 and TGF-β, and then find the key molecules which can regulate them by immunoprecipitation, Western blot, RT-qPCR, CHIP, nuclear and cytoplasmic separation test.

**Results:**

The results of the present study show that the abnormal activation of KRAS induced the overexpression of ITGA2 in pancreatic cancer. Moreover, ITGA2 expression significantly suppressed the activation of the TGF-β pathway. ITGA2 silencing enhanced the anti-pancreatic cancer proliferation and tumor growth effects of TGF-β. Mechanistically, ITGA2 expression suppressed the activation of the TGF-β pathway by inhibiting the SMAD2 expression transcriptionally. In addition, it interacted with and inhibited the nuclear translocation of TFCP2, which induced the SMAD2 expression as a transcription factor. Furthermore, TFCP2 also induced ITGA2 expression as a transcription factor, and the TFCP2 feedback regulated the ITGA2-TFCP2-SMAD2 pathway.

**Conclusions:**

Taken together, these results indicated that ITGA2 expression could inhibit the activation of the TGF-β signaling pathway in pancreatic cancer via the TFCP2-SMAD2 axis. Therefore, ITGA2, by effectively enhancing the anti-cancer effects of TGF- β, might be a potential clinical therapeutic target for pancreatic cancer.

**Supplementary Information:**

The online version contains supplementary material available at 10.1186/s13046-022-02286-5.

## Background

Pancreatic ductal adenocarcinoma is the seventh leading cause of cancer death worldwide and one of the deadliest solid tumors, with a 5-year survival rate of < 8%, which is further reduced by increased morbidity, late diagnosis, and poor treatment [1–3]. Although the efficacy and survival rates are better with combination therapy with cell inhibitors than with gemcitabine monotherapy, the therapeutic effect is still limited [2]. Elucidating the mechanisms underlying pancreatic cancer progression and identifying potential therapeutic targets are critical to the improvement of the prognosis of patients with pancreatic cancer.

Integrin is a cell surface receptor that mediates cell-to-cell adhesion and the cell and extracellular matrix [4]. Integrin α2 and β1 subunits form heterodimers of the transmembrane receptors, which are important molecules involved in tumor cell proliferation, migration, survival, and angiogenesis [5, 6]. Integrin α2/β1 is overexpressed in many kinds of cancer cells, but its expression is low or even nonexistent in most normal organs and tissues [6]. Integrin alpha 2 (ITGA2) encodes a subunit of transmembrane receptors for collagen and related proteins [7]. The protein encoded by the gene forms a heterodimer with a β subunit, which mediates the adhesion of platelets and other cell types to the extracellular matrix [6]. ITGA2 is overexpressed in many cancers such as hepatocellular carcinoma [8], ovarian cancer [9], and pancreatic ductal adenocarcinoma [10], and is thought to be related to cell adhesion and cell surface-mediated signal transduction. Although our previous studies showed that overexpressed ITGA2 can upregulate PD-L1 to activate the STAT3 signaling pathway and thereby promote tumor cell progression [10], the carcinogenic mechanism of ITGA2 in pancreatic cancer still needs elucidation in further research.

Our present study shows that overexpressed ITGA2 can inhibit the SMAD2 expression by interacting with and inhibiting the nuclear translocation of TFCP2, the transcription factor of SMAD2. Thus, ITGA2 overexpression can further inhibit the TGF-β pathway to promote the proliferation of pancreatic cancer cells. Therefore, our findings indicate that ITGA2 might become a new therapeutic target for pancreatic cancer, especially when combined with TGF-β treatment.

## Materials and methods

### Cell culture

Pancreatic cancer cell lines PANC-1 and AsPC-1 were purchased from American type culture collection (ATCC, USA). The cell lines were cultured in Dulbecco’s modified eagle medium (DMEM, Thermo Fisher Scientific, USA) supplemented with 10% fetal bovine serum (FBS, HyClone, USA) and incubated at 37 °C with 5% CO_2_ concentration.

### Antibodies, plasmids, and chemicals

Human expression vectors for the flag-tagged recombinant proteins were constructed using the pcDNA3.1 backbone vector. The antibodies for ITGA2 (#ab133557, Abcam, USA), GAPDH (#10494–1-AP, Proteintech, USA), TFCP2 (#15203-1-AP, Proteintech, USA), and SMAD2 (#13684S, Cell Signaling Technology, USA) and the recombinant proteins, including TGF-β (#ab50036; Abcam, USA), KRAS^G12D^ inhibitor (#S8499, SELLECK, USA), and U0126 (#HY-12031A; MedChemExpress, USA) recombinant proteins were purchased from the respective companies.

### RNA interference

Lipofectamine 3000 (Invitrogen, USA) and Opti-MEM media (Invitrogen, USA) were used for the transfection studies. For this purpose, 293 T cells were transfected with shRNA plasmids and viral packaging plasmids (pVSV-G and pEXQV) using lipofectamine 3000. After 24 h of transfection, the medium was replaced with fresh DMEM, containing 10% FBS and 1 mM sodium pyruvate. After 48 h of transfection, the virus culture medium was collected and added to the PANC-1 and AsPC-1 cells, which were supplemented with 12 μg/ml of polybrene. After 24 h of infection with virus culture medium, the infected PANC-1 and AsPC-1 cells were selected using 10 μg/ml of puromycin. The sequences of shRNAs are provided in Supplementary Table 1.

### Immunoprecipitation and Western blot analysis

Radioimmunoprecipitation assay (RIPA) lysis buffer, containing 1% protease inhibitor and phosphatase inhibitor was used for the cells’ lysis on ice and the cell lytic products were obtained. After centrifugation at 12000 rpm at 4 °C for 15 min, the undissolved impurities were removed and the supernatant was collected. The concentration of protein contents was measured using BCA’s experimental method. The protein extract was incubated overnight with agarose beads having antibodies in a slow-shaking incubator at 4 °C for co-immunoprecipitation test or Western blot analysis. The precipitated immune complexes were separated using sodium dodecyl sulfate polyacrylamide gel electrophoresis (SDS-PAGE) and transferred to a 0.45-μm polyvinylidene fluoride (PVDF) membrane. The membrane was then blocked with 0.5% bovine serum albumin (BSA) and incubated with the specific primary antibodies. The membrane was then visualized using the electrogenerated chemiluminescence (ECL) method. Bio-Rad microscopic imaging system was used to capture images, which were then analyzed using Image Lab software.

### Reverse transcription-quantitative polymerase chain reaction (RT-qPCR)

After total RNA extraction from the cells using TRIzol reagent (Invitrogen, 15,596,026, USA), the RNA concentration was determined using a spectrophotometer. The RNA samples (1 μg) were reverse-transcribed using a PrimeScript™ RT reagent Kit (TAKARA, RR047A, JPN). RT-qPCR was performed using a TB Green™ Fast qPCR Mix kit (TAKARA, RR430A, JPN). The data were presented as the average of three technical replicates from at least five independent experiments (biological replicates).

The primer sequences for the genes are provided in Supplementary Table 2.

### Liquid chromatography-tandem mass spectrometry (LC-MS/MS) analysis

The 293 T cells transfected with a Flag-PTEN-expressing plasmid were used for the identification of novel PTEN-binding proteins. The PTEN protein was immunoprecipitated using an anti-PTEN antibody and protein A + G agarose beads (#P2012, Beyotime, China) at 4 °C. LC-MS/MS analysis was performed using a Thermo Scientific Ultimate 3000 RSLC system combined with Q Exactive Plus high-resolution mass spectrometer by Shanghai Applied Protein Technology. The data were retrieved using MaxQuant (v1.6.6) software and the Andromeda algorithm. UniProt human proteome database was used as a reference database. The proteins and peptides with a false discovery rate (FDR) of 1% were selected.

### RNA sequencing

A total of 1 μg extracted RNA sample was used for RNA sequencing (RNA-seq) per sample. The sequencing libraries were generated using the NEBNext Ultra RNA Library Prep Kit for Illumina (NEB, USA), following the manufacturer’s instructions. The clustering of the samples was performed using the cBot Cluster Generation System with the TruSeq PE Cluster Kit v3-cBot-HS (Illumina), following the manufacturer’s instructions. After clustering, the libraries were sequenced on an Illumina NovaSeq platform, generating 150-bp paired-end reads. FeatureCounts v1.5.0-p3 software was used for counting the read numbers mapped to each gene. Differential expression analysis (two biological replicates per condition) was performed using the DESeq2 R package (1.16.1). ClusterProfiler R package was used to test the statistical enrichment of differentially expressed genes (DEGs) in Kyoto Encyclopedia of Genes and Genomes (KEGG) pathways.

### Colony formation assay

Colony formation assay was used for detecting the biological effects of ITGA2 and TGF-β on the survival of tumor cells. Four stable PANC-1 and AsPC-1 cell lines groups, including sh-Control, sh-ITGA2, TGF- β-treated sh-Control, and TGF- β-treated sh-ITGA2, were inoculated into six-well plates with 500 cells per well; all the cell lines groups were set up in replicates. After 10-14 days of culture, the colony formation assay was fixed, stained, and photographed.

### Cell counting Kit-8 (CCK8) cell proliferation assay

The four stable cell lines were used for four treatment groups, including control and experimental groups, and five replicates were set up for each group. A total of 2000 cells and 10 μL of CCK8 solution were added to each well. The cells were incubated in the incubator for 3 h and the absorbance at 450 nm was measured using an enzyme labeling instrument. The data were monitored continuously for 5 days and the final data were processed using GraphPad Prism v7.

### Chromatin Immunoprecipitation (ChIP) assay

The binding sites of ITGA2 and SMAD2 with TFCP2 were determined using a CHIP assay. For this purpose, formaldehyde was added to the cells for cross-linking the target proteins with genomic DNA and digesting the cells to form lysate. These samples were then treated with ultrasound to break the genomic DNA into 200-1000 bp fragments. The target proteins and the DNA fragments bound to them were co-immunoprecipitated, purified, and amplified using PCR. The primer sequences for ChIP-qPCR are provided in Supplementary Table 3.

### Nuclear-cytoplasmic separation

Two transfected cell lines (sh-control and sh-ITGA2) were collected in a centrifuge tube, to which, the cytoplasmic protein extraction reagent A with pre-added phenylmethanesulfonyl fluoride (PMSF) was added. After shaking and mixing, the cytoplasmic protein extraction reagent B was added to the samples and incubated on ice for 10 min. After shaking and mixing, the samples were centrifuged at 12000 g and 4 °C for 5 min. The supernatants were collected in the precooled centrifuge tubes and the cytoplasmic proteins were obtained, while the pellets were collected for nuclear proteins extraction. The nuclear protein extraction reagent with pre-added PMSF was added to the pellet. The samples were then incubated in the ice bath for 2, followed by shaking for 20 s; this process was repeated for a total of 30 min. The above samples were then centrifuged at 1000 g, 4 °C for 10 min, and the supernatants were obtained as nuclear proteins. The concentrations of cytoplasmic and nuclear proteins were measured using BCA. Finally, a Western blot analysis was carried out.

### Flow cytometry

The four stable pancreatic cancer cell lines were digested with trypsin in their logarithmic growth phase and collected in a flow tube with a pre-added culture medium. After centrifugation at 300 g for 5 min, the supernatants were discarded. After washing with PBS, the pellets were re-suspended in 300 μL of binding buffer, to which, 5 μL of Annexin V (FITC) was added and incubated in dark for 10 min. Then, 5 μL of propidium iodide (PI) was added, mixed, and incubated in dark for 5 min. The corresponding channels were detected and observed within 1 h.

### Bioinformatics mining

The correlations among TFCP2, ITGA2, and SMAD2 were analyzed using GEPIA (http://gepia.cancer-pku.cn/) database. The binding sites of TFCP2 with ITGA2 and SMAD2 were predicted using EPD (https://epd.epfl.ch//index.php). The pathway enrichment analysis of the RNA-Seq results was performed using R language and gene set enrichment analysis (GSEA) analysis. The expression patterns of ITGA2 in the KRAS wild-type and mutant pancreatic cancer cells were obtained using the cBioPortal database (https://www.cbioportal.org/) query.

### Mouse tumor model

All the animal experiments were performed following the guidelines of the Ethics Committee of Tongji Medical College, Huazhong University of Science and Technology. Approximately, 5 × 10^6^ PANC-1 cells infected with either sh-Control or sh-ITGA2 were injected subcutaneously into the left side of BALB/c-nu mice (4-5 weeks old male mice). The intra-tumoral injection of TGF-β recombinant protein was performed 3 times on days 1, 4, and 7 at a dose of 10 mg/kg [11]. The tumor size was measured using a digital vernier caliper every 2 days and the tumor volumes were calculated using the formula (L x W^2^)/2. The mice were sacrificed on day 21 or when tumor volume reached 1000 mm^3^.

### Immunofluorescence and immunohistochemistry staining

The cells were divided into two groups, including the treatment group (sh-ITGA2) and the control group (sh-control). The tissue sections were fixed with 4% PFA for 20 min at room temperature. After washing with PBS, the tissue sections were sequentially transferred to Triton-X-100 and 5% BSA solutions for 30 min, respectively. After washing three times with PBS for 5 min each, the tissue sections were incubated with rabbit anti-ITGA2 antibody and rabbit anti-TFCP2 antibody at a concentration of 1:1000 for 16 h. The sections were then rinsed with PBS three times and incubated with the respective fluorochrome-conjugated secondary antibodies for 1 h at room temperature. DAPI (4′,6-diamidino-2-phenylindole) counterstaining was performed to stain the nuclei. The primary antibodies used included anti-ITGA2 antibody (1:200 dilution; Abcam) and anti-TFCP2 antibody (1:200 dilution; Proteintech). For studying the immunohistochemistry, the tumor tissue sections were dehydrated with ethanol, washed with xylene, and mounted, followed by immunostaining of the sections in replicates. In order to quantify the status of Ki67 protein expression, each tissue section was visualized and the average percentage of positive cells was determined in at least 5 random fields. Fluorescence images were obtained using an immunofluorescent microscope (Olympus IX71, Japan) and a confocal laser-scanning microscope (LSM780, ZEISS, Germany).

### Statistical analyses

All the experiments were performed in at least three replicates. Parametric data are shown as means ± standard errors of the mean (SEMs), while the nonparametric data are shown as medians and ranges. Two-way or one-way analysis of variance (ANOVA) with Tukey’s multiple comparison test was used for the multiple group analysis. Unpaired Student’s *t*-tests were used to compare the data between two groups. Two-tailed *P*-values < 0.05 were considered statistically significant. All the statistical analyses were performed using GraphPad Prism 6 software (GraphPad Software, Inc., USA).

## Results

### Abnormal KRAS activation induced the overexpression of ITGA2 in pancreatic cancer cells

**Fig. 1:**
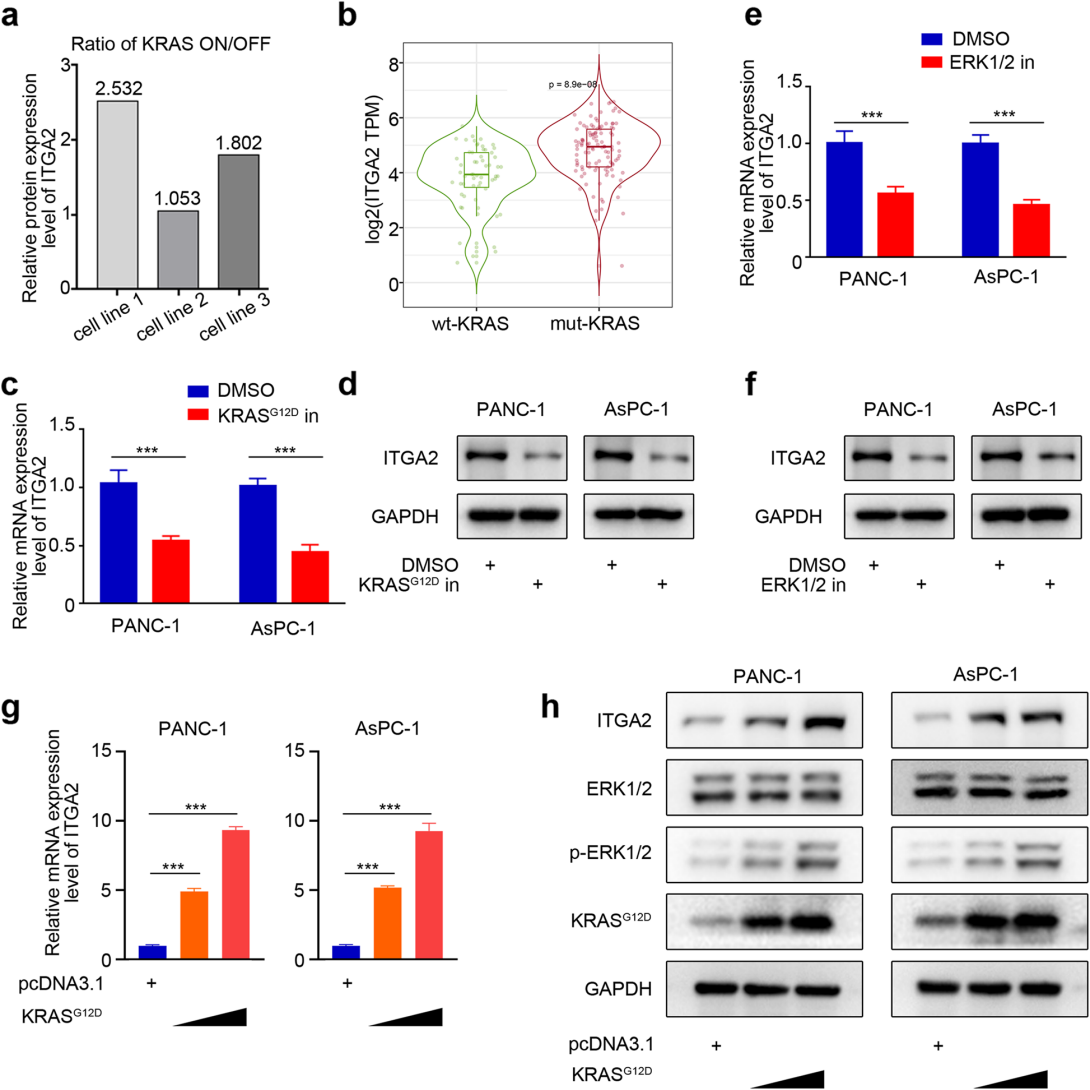
Abnormal KRAS activation induced the overexpression of ITGA2 in pancreatic cancer cells. **a** ITGA2 is one of the KRAS up-regulated surfaceome in different pancreatic ductal adenocarcinoma (PDAC) cell lines (AK10965, AK192, AK196) [16]. **b** Cancer molecular expression profile data of cBioPortal, showing the expression of ITGA2 in wt-KRAS and mut-KRAS pancreatic cancer. **c** RT-PCR was used to determine the mRNA expression level of *ITGA2* in PANC-1 and AsPC-1 cells treated with KRAS^G12D^ inhibitor (KRpep-2d, 10uM). *GAPDH* served as an internal reference and repeated in triplicates. ***P < 0.001. **d** Western blot analysis for determining the protein expression level of ITGA2 in the PANC-1 or AsPC-1 cells treated with KRAS^G12D^ inhibitor (KRpep-2d, 10uM). GAPDH served as an internal reference. **e** RT-PCR was used to determine the expression level of *ITGA2* in PANC-1 and AsPC-1 cells treated with ERK 1/2 inhibitors (U0126, 10uM). *GAPDH* served as an internal reference and repeated in triplcates. ***P < 0.001. **f** Western blot analysis for detrmining the protein expression level of ITGA2 in the PANC-1 or AsPC-1 cells treated with ERK1/2 inhibitor (U0126, 10uM). GAPDH served as an internal reference. **g** RT-PCR was used to determine the expression level of *ITGA2* in PANC-1 and AsPC-1 cells infected with KRAS^G12D^ plasmids. *GAPDH* served as an internal reference and repeated in triplcates. ***P < 0.001. **h** Western blot analysis for determining the protein expression level of ITGA2, ERK1/2, p-ERK1/2, KRAS in the PANC-1 or AsPC-1 cells infected with KRAS^G12D^ plasmids. GAPDH served as an internal reference

The KRAS-mediated RAS signaling pathway plays an important role in the occurrence and development of diseases and resistance to the chemotherapeutic drug. *KRAS* is one of the main driving genes in pancreatic cancer. More than 90% of pancreatic cancer patients have *KRAS* carcinogenic point mutations, which constitutively activate the RAS signaling pathway [12–14]. The RAS signal transduction regulates multiple cellular biological processes in the pancreatic ductal adenocarcinoma, including the proliferation, migration, metabolism, and autophagy of the cells [15]. In their study, Yao et al. found that the oncogenic *KRAS* mutations upregulated the ITGA2 protein on the cell surface [16] (Fig. 1a). Consistent with this finding, searching the cBioPortal database showed that the ITGA2 expression level significantly increased in pancreatic cancer with *KRAS* mutation (Fig. 1b). In order to confirm this, the pancreatic cancer cells were treated with KRAS^G12D^ or ERK1/2 inhibitors. The RT-PCR and Western blot analysis showed that the ITGA2 expression was significantly inhibited in the pancreatic cancer cells treated with KRAS^G12D^ or ERK1/2 inhibitors (Fig. 1c–f). In order to explore the accuracy of this conclusion and the regulatory mechanism of KRAS on ITGA2, the plasmid was transfected into the pancreatic cancer cell lines to overexpress KRAS^G12D^. The mRNA and protein levels of KRAS^G12D^ were detected using RT-PCR and Western blot analysis, which showed that the overexpression of KRAS^G12D^ increased the phosphorylation level of ERK1/2, further upregulating the expression of ITGA2 (Fig. 1g and h). This might be a possible regulatory mechanism of KRAS on ITGA2. Therefore, these results indicated that the abnormal activation of KRAS induced the overexpression of ITGA2 in the pancreatic cancer cells.

### *ITGA2* silencing activated the TGF-β signaling pathway in pancreatic cancer cells

**Fig. 2:**
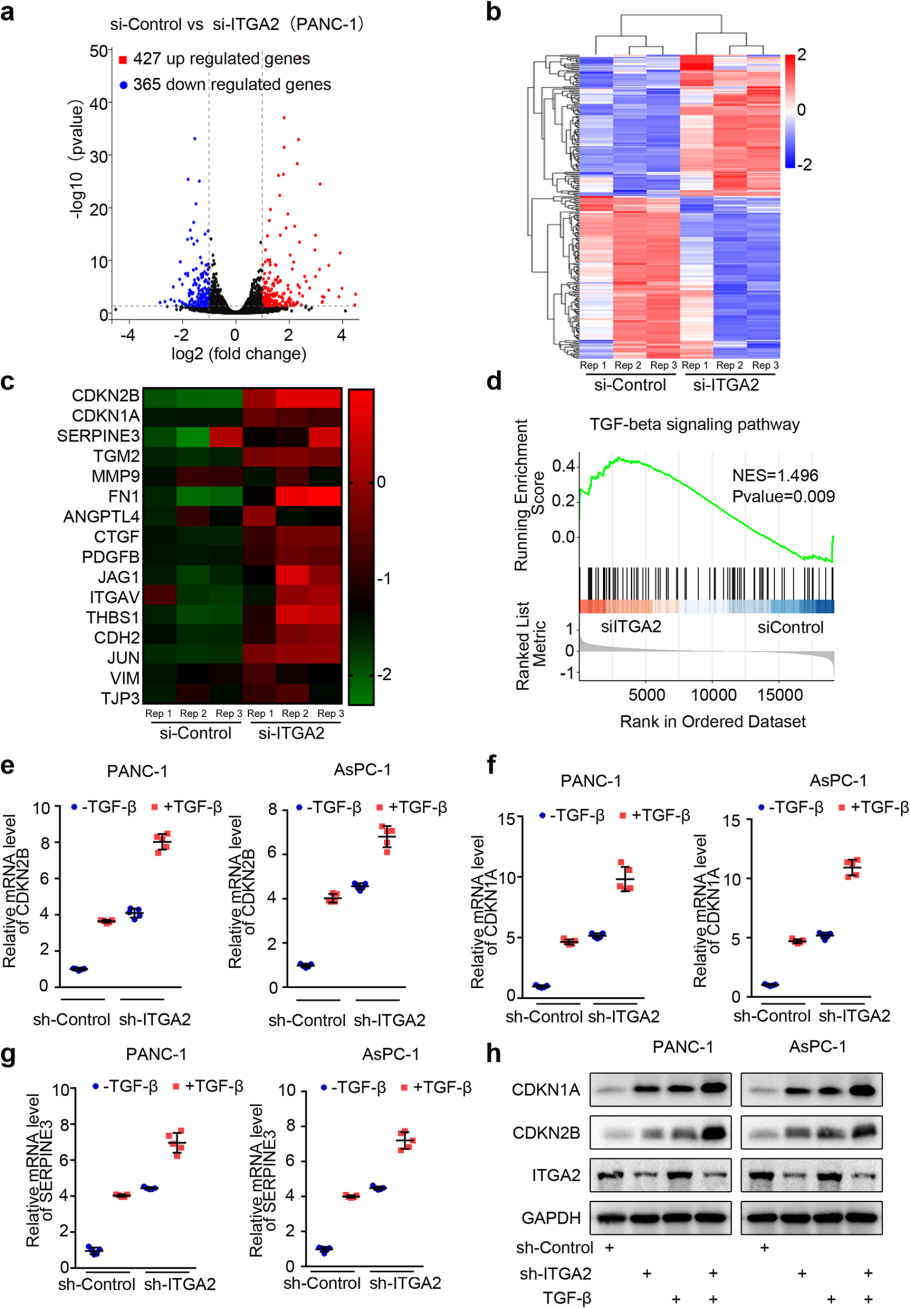
*ITGA2* silencing activated TGF-β signaling pathway in pancreatic cancer cells. **a** and **b** Volcano plot (**a**) and heatmap (**b**) showing the differentially expressed genes in PANC-1 cells infected with si-Control or si-ITGA2. The blue points represent the downregulated genes (*n* = 365), while the red points represent the upregulated genes (*n* = 427). **c** Heatmap showing a group of *ITGA2* knockdown-regulated genes in the TGF-β signaling pathway. **d** Gene set enrichment analysis (GSEA) was performed using R package. Genes associated with the TGF-β signaling pathway in the si-ITGA2 group were significantly enriched as compared to the si-control group. *P* = 0.009. **e-g.** PANC-1 and AsPC-1 cells infected with sh-ITGA2 or sh-Control were treated with or without TGF-β recombinant protein for 24 h (5 ng/ml). RT-PCR was used to detect the mRNA expression levels of the downstream genes in the TGF-β signaling pathway, including *CDKN2B* (**e**), *CDKN1A* (**f**), and *SERPINE3* (**g**). GAPDH served as an internal reference and repeated in triplicates. ***P < 0.001. **h** Western blot analysis was used to detect the protein level of CDKN1A, CDKN2B, and ITGA2 in the PANC-1 and AsPC-1 cells

Based on previous studies, the present study verified that the overexpression of ITGA2 could promote the proliferation and invasion of tumor cells by regulating the PD-L1 expression [10]. In order to further study the regulatory mechanism of ITGA2 expression on the progression of pancreatic cancer cells, the previous RNA-sequencing data was reanalyzed [10] and identified 427 upregulated and 365 downregulated DEGs (Fig. 2a and b). In addition, the KEGG and GSEA showed that the TGF-β was significantly activated after silencing the *ITGA2*, indicating that the ITGA2 expression could inhibit the activation of the TGF-β signaling pathway (Fig. 2c and d). Moreover, the RT-PCR results showed that the silencing of *ITGA2* in the pancreatic cancer cells significantly increased the mRNA levels of cyclin dependent kinase inhibitor 2B (*CDKN2B*), cyclin dependent kinase inhibitor 1A (*CDKN1A*), and serpin family E member 3 (*SERPINE3*), which are the downstream molecules of the TGF-β signaling pathway (Fig. 2e–g). Furthermore, the silencing of *ITGA2* further increased the expression levels of downstream molecules in the TGF-β signaling pathway induced by TGF-β recombinant protein (Fig. 2e–g). Western blot analysis (Fig. 2h) showed the protein levels of the downstream molecules in the TGF-β signaling pathway were consistent with the RT-PCR results. Therefore, these results showed that the silencing of *ITGA2* could activate the TGF-β signaling pathway in pancreatic cancer cells.

### ITGA2 silencing enhanced the anti-pancreatic cancer cell proliferation effect of TGF-β

**Fig. 3:**
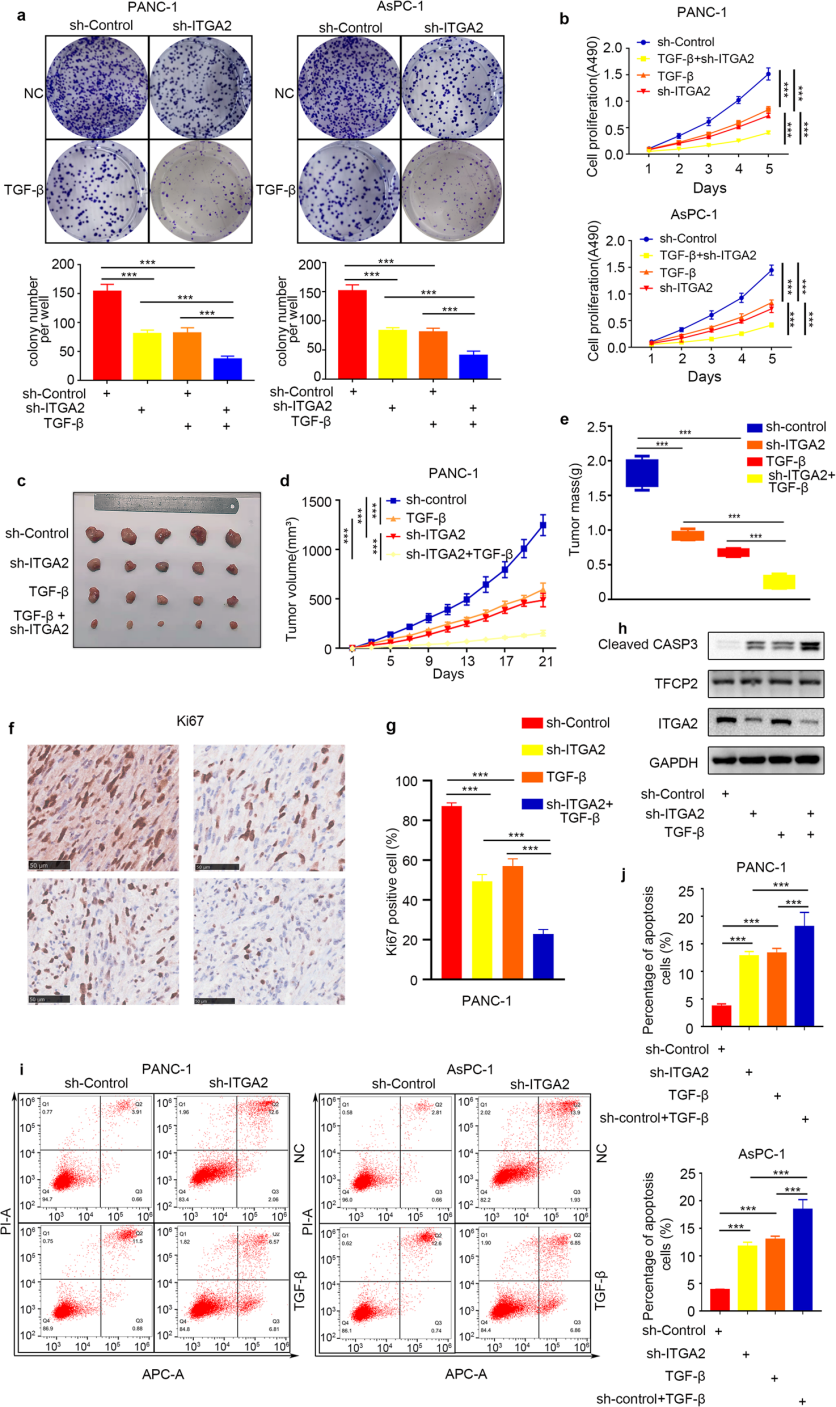
*ITGA2* silencing enhanced the anti-pancreatic cancer cells proliferation effect of TGF-β. **a** and **b** PANC-1 and AsPC-1 cells, infected with sh-ITGA2 or sh-Control, were harvested for colony formation assay (**c**) and CCK-8 cell proliferation assay (**d**). Each bar represents the mean ± SD of the three independent replicates. ***P < 0.001. **c-e** PANC-1 cells, infected with sh-ITGA2 or sh-Control, were subcutaneously injected into the nude mice and treated with or without TGF-β recombinant protein (10 mg/kg). The tumors tissues were collected and photographed on day 21(**c**). The data for tumor volume (**e**) and tumor mass (**f**) are shown as the mean ± SD (*n* = 5). ***P < 0.001. **f** and **g** Tumor tissues collected from nude mice were used for immunohistochemistry (**f**) to show the number of Ki67 positive cells (**g**). Each bar represents the mean ± SD of the five independent experiments. ***P < 0.001. **h** Protein extracted from tumors, which were collected from nude mice, were used to detected the expression of cleaved CASP3, TFCP2, and ITGA2 using Western blot analysis. **i** and **j** PANC-1 and AsPC-1 cells, infected with sh-Control or sh-ITGA2, were harvested for Annexin V-FITC/PI dual staining assay using flow cytometry (**f**) and the percentage of apoptotic cells was qualified (**g**). The data are shown as means with error bars, representing SD (*n* = 3). ***P < 0.001

Considering that ITGA2 silencing activated the TGF-β signaling pathway in pancreatic cancer cells, it was hypothesized that the *ITGA2* silencing might enhance the anti-pancreatic cancer cell proliferation effect of TGF-β. In order to verify this, the PANC-1 and AsPC-1 cells were transfected with sh-ITGA2, sh-Control, or TGF-β to detect their biological functions. The CCK-8 cell proliferation and colony formation assay showed that the ITGA2 silencing significantly could inhibit the proliferation ability of pancreatic cancer cells (Fig. 3a and b). In addition, the *ITGA2* silencing and TGF-β expression also promoted the apoptosis of pancreatic cancer cells and their combination increased the apoptosis rate of tumor cells (Fig. 3i and j). In order to investigate the biological effects of *ITGA2* silencing and TGF-β expression on pancreatic cancer in-vivo, the PANC-1 cells with or without *ITGA2* gene silencing were subcutaneously injected into the nude mice to induce tumorigenesis. The tumor volumes were measured every 4 days. After 21 days, the tumors were photographed and weighed. Finally, the tumor mass and volume were statistically analyzed. As shown in Fig. 3c–e, the tumors formed in the *ITGA2*-silenced PANC-1 cells and the TGF-β-treated PANC-1 cells were smaller and lighter as compared to the controls, whereas those formed in the mice treated with the combined *ITGA2* silencing and TGF-β treatment were smaller and lighter as compared to their individual treatment. In order to assess the difference in apoptosis and cell growth in the in-vivo experiments, the expressions of ITGA2 (Fig. 3h), proliferation marker Ki-67 (Fig. 3f and g), and apoptosis marker cleaved-Cacpase3 (Fig. 3h) were detected in the collected mice tumors. The results suggested that the *ITGA2* silencing and/or TGF-β treatment significantly inhibited the Ki-67 expression and induced the expression of cleaved-Cacpase3 in the collected mice tumors. This indicated that ITGA2 could promote the proliferation of tumor cells and inhibit their apoptosis in-vivo. Therefore, it was concluded that the ITGA2 silencing enhanced the anti-pancreatic cancer cell proliferation effect of TGF-β.

### *ITGA2* silencing induced the SMAD2 expression in pancreatic cancer cells

**Fig. 4:**
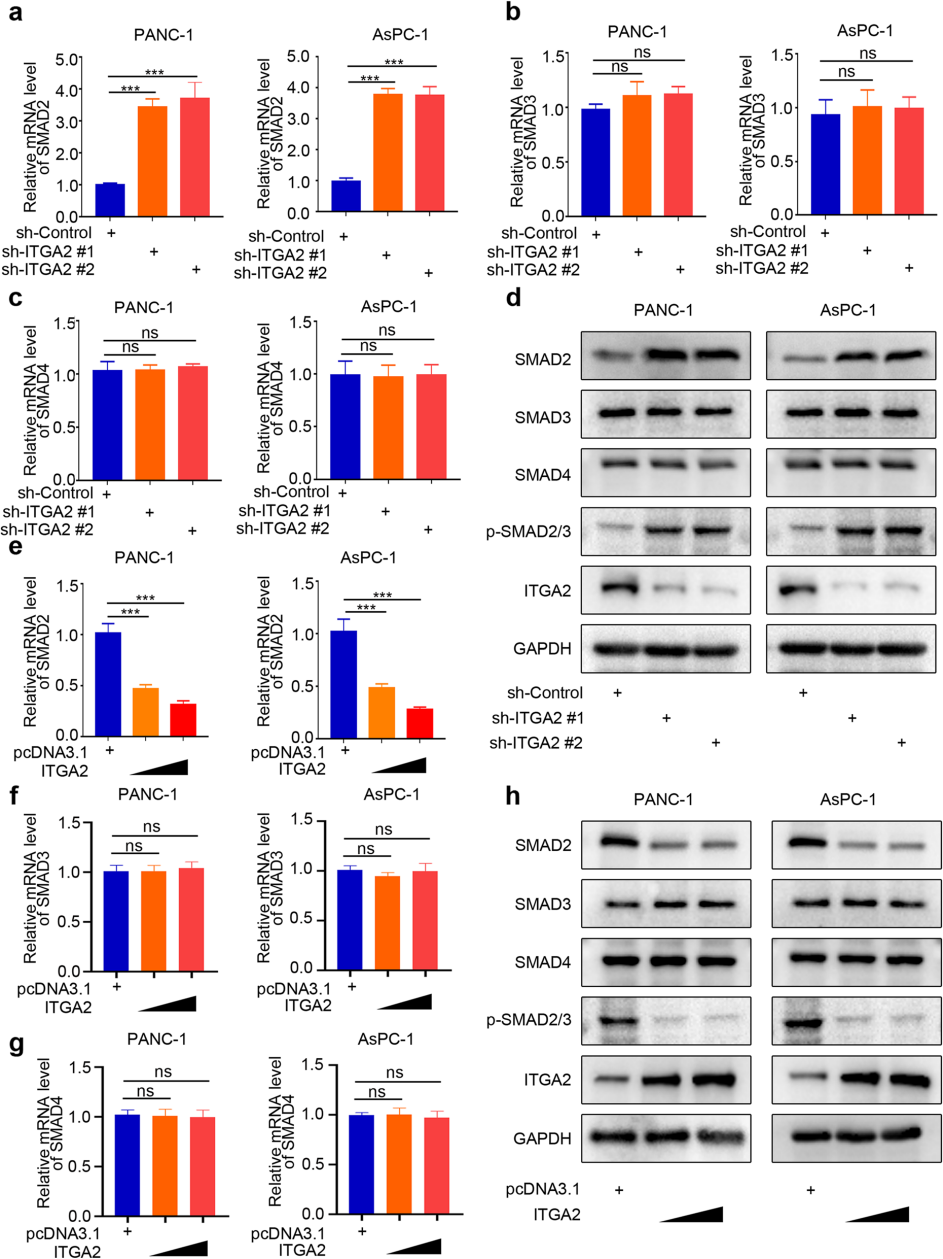
*ITGA2* silencing induced the expression of SMAD2 in pancreatic cancer cells. **a-c** RT-PCR was used to determine the mRNA expression level of *SMAD2* (**a**), *SMAD3* (**b)**, and *SMAD4* (**c**) in the PANC-1 and AsPC-1 cells infected with sh-Control or sh-ITGA2s. *GAPDH* served as an internal reference and repeated in triplicates. ns, not significant; ***P < 0.001. **d** Western blot analysis was used to determine the protein expression level of SMAD2, SMAD3, SMAD4, and p-SMAD2/3 in the PANC-1 and AsPC-1 cells infected with sh-Control or sh-ITGA2s. GAPDH served as an internal reference. **e-g** RT-PCR was used to determine the mRNA expression level of *SMAD2* (**e**), *SMAD3* (**f**) and *SMAD4* (**g**) in PANC-1 and AsPC-1 cells infected with or without ITGA2 plasmids. *GAPDH* served as an internal reference and repeated in triplicates. ***P < 0.001. **h** Western blot analysis was used to determine the protein expression level of SMAD2, SMAD3, SMAD4, and p-SMAD2/3 in the PANC-1 and AsPC-1 cells infected with or without ITGA2 plasmids. GAPDH served as an internal reference

The activation of the TGF-β signaling pathway requires an entry of the complex formed by SMAD2 and SMAD3 with SMAD4 into the nucleus [17]. Therefore, it was speculated that the *ITGA2* silencing might promote the activation of the TGF-β pathway by regulating the expression levels of SMAD2/SMAD3/SMAD4. The RT-PCR results revealed that the *ITGA2* silencing significantly increased the expression of *SMAD2* (Fig. 4a), but not those of the *SMAD3* or *SMAD4* (Fig. 4b and c). Meanwhile, the protein expression levels of SMAD2, SMAD3, SMAD4, and p-SMAD2/3 were also detected (Fig. 4d and Supplementary Fig. 1c) in the pancreatic cancer cells with the *ITGA2* gene silenced. The results showed that the *ITGA2* silencing could up-regulate the expression of SMAD2 and phosphorylation p-SMAD2/3, but not those of the SMAD3 or SMAD4. Furthermore, the overexpression of ITGA2 could significantly inhibit the mRNA level of *SMAD2* in pancreatic cancer cells, but not those of the *SMAD3* or *SMAD4* (Fig. 4e-g). Similarly, the overexpression of ITGA2 could inhibit the expression of *SMAD2* and phosphorylation of SMAD2/3 (Fig. 4h and Supplementary Fig. 1d) in the pancreatic cancer cell. Taken together, these findings indicated that the *ITGA2* silencing induced the SMAD2 expression in pancreatic cancer cells.

### ITGA2 inhibited the activation of the TGF-β pathway via the SMAD2 expression in pancreatic cancer cells

**Fig. 5:**
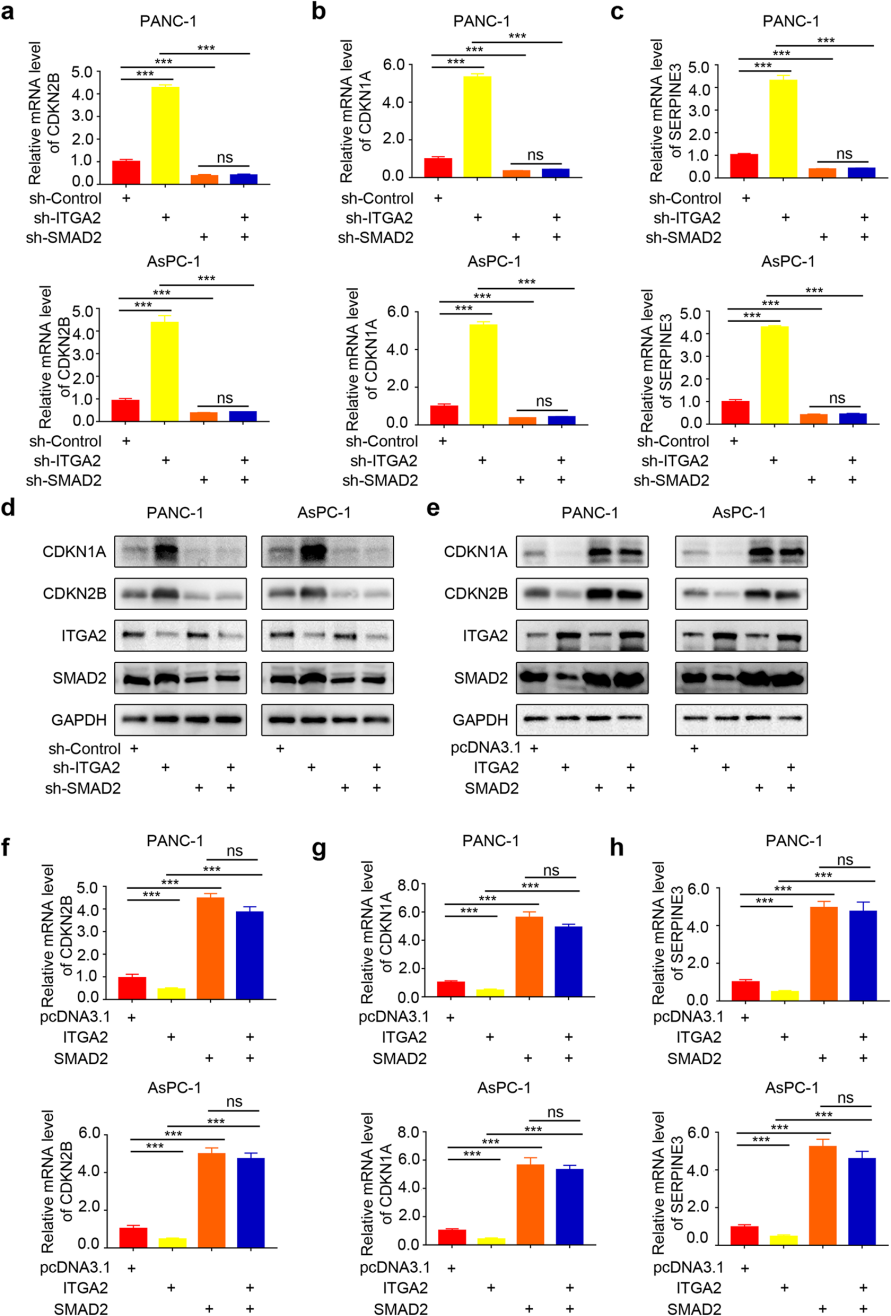
ITGA2 inhibited the activation of TGF- β pathway via SMAD2 in pancreatic cancer cells. **a-c** RT-PCR was used to determine the mRNA expression level of *CDKN2B* (**a**), *CDKN1A* (**b**) and *SERPINE3* (**c**) in the PANC-1 and AsPC-1 cells infected with sh-ITGA2 and/or sh-SMAD2. *GAPDH* served as an internal reference and repeated in triplicates. ns, not significant; ***P < 0.001. **d** Western blot analysis was used to determine the protein expression level of CDKN1A and CDKN2B in the PANC-1 and AsPC-1 cells infected with sh-ITGA2 and/or sh-SMAD2. GAPDH served as an internal reference and repeated in triplicates. ns, not significant; ***P < 0.001. **e** Western blot analysis was used to determine the protein expression level of CDKN1A and CDKN2B in the PANC-1 and AsPC-1 cells infected with ITGA2 plasmids and/or SMAD2 plasmids. GAPDH served as an internal reference and repeated in triplicates. ns, not significant; ***P < 0.001. **f-h** RT-PCR was used to determine the mRNA expression level of *CDKN2B* (**a**), *CDKN1A* (**b**) and *SERPINE3* (**c**) in the PANC-1 and AsPC-1 cells infected with ITGA2 plasmids and/or SMAD2 plasmids. *GAPDH* served as an internal reference and repeated in triplicates. ns, not significant; ***P < 0.001

In order to verify whether SMAD2 was the key molecule of the TGF-β signaling pathway regulated by ITGA2, the expression levels of the downstream genes in the TGF-β signaling pathway, including *CDKN2B*, *CDKN1A*, and *SERPINE3*, were detected. The RT-PCR results (Fig. 5a-c) and Western blot analysis (Fig. 5d) showed that the *SMAD2* silencing could reverse the upregulation of *CDKN2B*, *CDKN1A*, and *SERPINE3* gene expressions induced by *ITGA2* silencing. In contrast, the *SMAD2* overexpression reversed the downregulation of the *CDKN2B*, *CDKN1A*, and *SERPINE3* gene expressions induced by the *ITGA2* overexpression (Fig. 5e-h). Besides, the biological functions of these proteins were detected using the colony formation assay (Supplementary Fig. 1a) and CCK8 cell proliferation assay (Supplementary Fig. 1b) in pancreatic cancer cells with *ITGA2* and/or *SMAD2* genes silenced. The results showed that *SMAD2* gene silencing could reverse the biological function conferred by *ITGA2* depletion. Furthermore, the *SMAD3* gene in *ITGA2*-silenced cells was knocked down and the mRNA and protein levels of the downstream molecules of the TGF-β signaling pathway were detected, which showed the reversal effect of *SMAD3* was similar to that of the *SMAD2* (Supplementary Fig. 2a-d). However, the reversal effect of *SMAD3* was not as obvious as that of *SMAD2*. This was attributed to the complex formation of SMAD2 and SMAD3 in the TGF-β pathway, which then entered the nucleus to transmit signals [18]. Therefore, these results showed that ITGA2 inhibited the activation of the TGF-β pathway via the SMAD2 expression in the pancreatic cancer cells.

### ITGA2 inhibited the SMAD2 expression by interacting with TFCP2 in pancreatic cancer cells

**Fig. 6:**
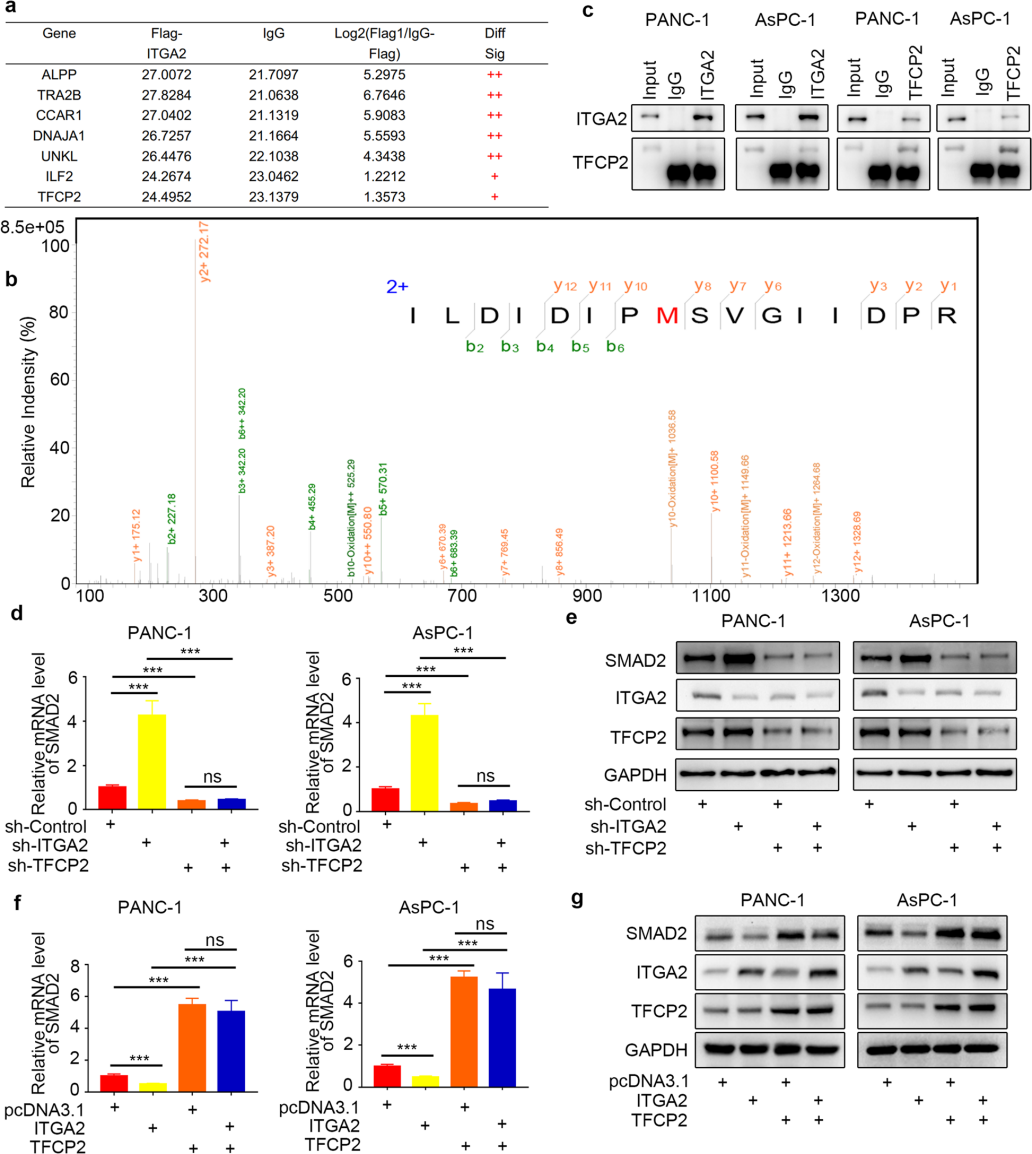
ITGA2 inhibited the expression of SMAD2 by interacting with TFCP2 in pancreatic cancer cells. **a-b** LC-MS/MS was used to identify the interaction of TFCP2 with ITGA2 (**a**) by detecting the peptided of TFCP2 (**b**). **c** Immunoprecipitation assay, showing the interaction between ITGA2 and TFCP2 in the PANC-1 and AsPC-1 cells. **d** RT-PCR was used to determine the mRNA expression level of *SMAD2* in the PANC-1 and AsPC-1 cells infected with sh-ITGA2 and/or sh-TFCP2. *GAPDH* served as an internal reference and repeated in triplicates. ns, not significant; ***P < 0.001. **e** Western blot analysis was used to determine the protein expression level of SMAD2 in the PANC-1 and AsPC-1 cells infected with sh-ITGA2 and/or sh-TFCP2. GAPDH served as an internal reference. **f** RT-PCR was used to determine the mRNA expression level of *SMAD2* in PANC-1 and AsPC-1 cells infected with ITGA2 plasmids and/or TFCP2 plasmids. *GAPDH* served as an internal reference and repeated in triplicates. ns, not significant; ***P < 0.001. **g** Western blot analysis was used to determine the protein expression level of SMAD2 in the PANC-1 and AsPC-1 cells infected with ITGA2 plasmids and/or TFCP2 plasmids. GAPDH served as an internal reference

The inhibitory effect of ITGA2 on the TGF-β signaling pathway by regulating the SMAD2 expression had been previously established, but the specific mechanism remained unclear. The LC-MS/MS analysis showed that the TFCP2 could interact with ITGA2; this interaction was identified by detecting the peptides of TFCP2 (Fig. 6a and b), which had been verified using immunoprecipitation assays in the pancreatic cancer cells (Fig. 6c). Therefore, it was supposed that the ITGA2 could inhibit the SMAD2 expression via TFCP2 in the pancreatic cancer cells. The RT-PCR assay and Western blot analysis showed that the *TFCP2* silencing reversed the upregulation of the *SMAD2* expression induced by *ITGA2* silencing (Fig. 6d and e), while TFCP2 overexpression reversed the downregulation of SMAD2 expression, which was induced by the ITGA2 overexpression (Fig. 6f and g). Taken together, these results indicated that the ITGA2 could inhibit the SMAD2 expression by interacting with TFCP2 in the pancreatic cancer cells.

### TFCP2 induced the SMAD2 expression by acting as a transcription factor in the pancreatic cancer cells

**Fig. 7:**
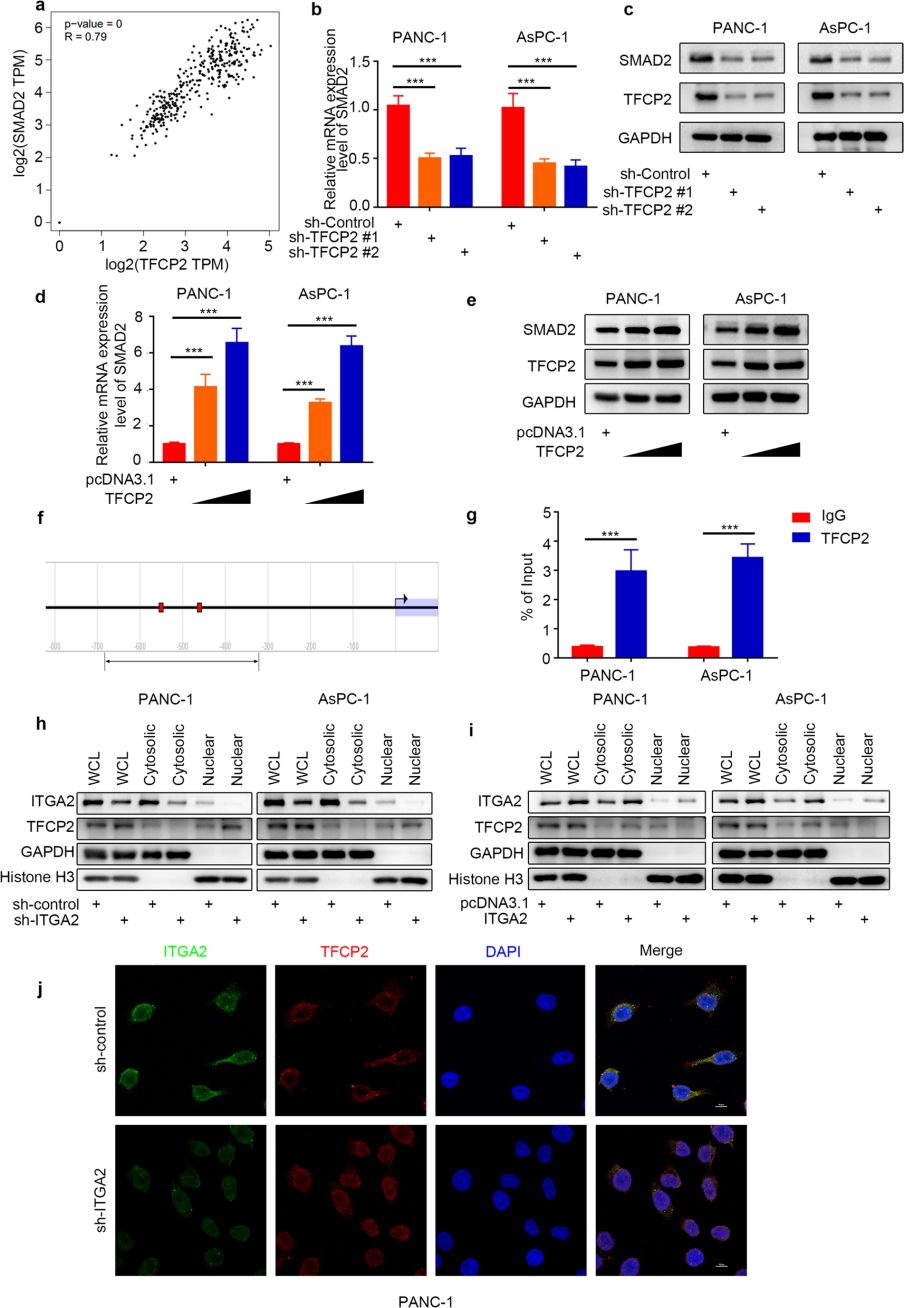
TFCP2 induced the expression of SMAD2 by acting as a transcription factor in pancreatic cancer cells. **a** GEPIA database was searched to determine the correlation between the mRNA expression levels of *SMAD2* and *TFCP2* in the PDAC samples. *P-* and *R*-values are indicated in the figure. **b** RT-PCR was used to determine the mRNA expression level of *SMAD2* in PANC-1 and AsPC-1 cells infected with sh-Control or sh-TFCP2s. *GAPDH* served as an internal reference and repeated in triplicates. ns, not significant; ***P < 0.001. **c** Western blot analysis was used to determine the protein expression level of SMAD2 in the PANC-1 and AsPC-1 cells infected with sh-Control or sh-TFCP2s. GAPDH served as an internal reference. **d** RT-PCR was used to determine the mRNA expression level of *SMAD2* in the PANC-1 and AsPC-1 cells infected with pcDNA3.1 or TFCP2 plasmids. *GAPDH* served as an internal reference and repeated in triplicates. ns, not significant; ***P < 0.001. **e** Western blot analysis was used to determine the protein expression level of SMAD2 in the PANC-1 and AsPC-1 cells infected with pcDNA3.1 or TFCP2 plasmids. GAPDH served as an internal reference. **f** EPD database was searched to determine the potential binding sites of TFCP2 in the promoter region in *SMAD2* gene. **g** ChIP-qPCR of *TFCP2* in the PANC-1 and AsPC-1 cells. All the data are shown as the mean values ± SD from three independent replicates. ***P < 0.001. **h and i** Western blot analysis was used to detect the protein expression level of TFCP2 in the cytoplasm and nucleus of PANC-1 cells and AsPC-1 cells infected with sh-Control or sh-ITGA2 (**h**) and pcDNA3.1 or ITGA2 plasmids (**i**). **j** Pancreatic cancer cells were divided into two groups, including treatment (sh-ITGA2) and control (sh-control) groups. The tissue sections were incubated with rabbit anti-ITGA2 antibody and rabbit anti-TFCP2 antibody for immunofluorescent to find the nuclear translocation of TFCP2

The present study previously found that the ITGA2 could inhibit the SMAD2 expression by interacting with TFCP2 in the pancreatic cancer cells (Fig. 6). However, the specific inhibitory mechanism was still unclear. Searching the GEPIA database showed that the mRNA expression levels of *TFCP2* were positively correlated with those of the SMAD2 in pancreatic cancer cells (Fig. 7a). The RT-PCR and Western blot analyses showed that the *TFCP2* silencing could significantly inhibit the transcription of the *SMAD2* gene in the pancreatic cancer cells (Fig. 7b and c). In addition, the RT-PCR and Western blot analyses also revealed that the *TFCP2* overexpression significantly promoted the transcription of the *SMAD2* gene in pancreatic cancer cells (Fig. 7d and e; Supplementary Fig. 1e). Studies have reported that TFCP2 could regulate the expressions of several targeted genes by acting as a transcription factor [19]. Searching the Eukaryotic Promoter Database (EPD) showed two potential binding sites for TFCP2 in the promoter region of the *SMAD2* gene (Fig. 7f), which were confirmed using a ChIP-PCR assay (Fig. 7g). ITGA2 inhibited the SMAD2 expression by interacting with TFCP2 (Fig. 6) but did not change the TFCP2 expression in the pancreatic cancer cells (Fig. 3h). Therefore, it was speculated that the ITGA2 inhibited the entry of transcription factors into the nucleus by interacting with TFCP2, thereby inhibiting the expression of the *SMAD2* gene. In order to verify this conjecture, the separation of nuclear and cytoplasmic separation proteins (Fig. 7h and i) and immunofluorescence (Fig. 7j) experiments were conducted in the *ITGA2* silenced and *ITGA2* normal-expressing PANC-1 cells for depicting the nuclear translocation of TFCP2 upon ITGA2 depletion. The results showed that the *ITGA2* silencing significantly induced the nuclear translocation of TFCP2. Taken together, these findings indicated that TFCP2 induced the SMAD2 expression by acting as a transcription factor in the pancreatic cancer cells. The ITGA2 inhibited the SMAD2 expression by interacting with TFCP2 and inhibiting its nuclear translocation in the pancreatic cancer cells.

### TFCP2 as transcription factor feedback induced the ITGA2 expression in pancreatic cancer cells

**Fig. 8:**
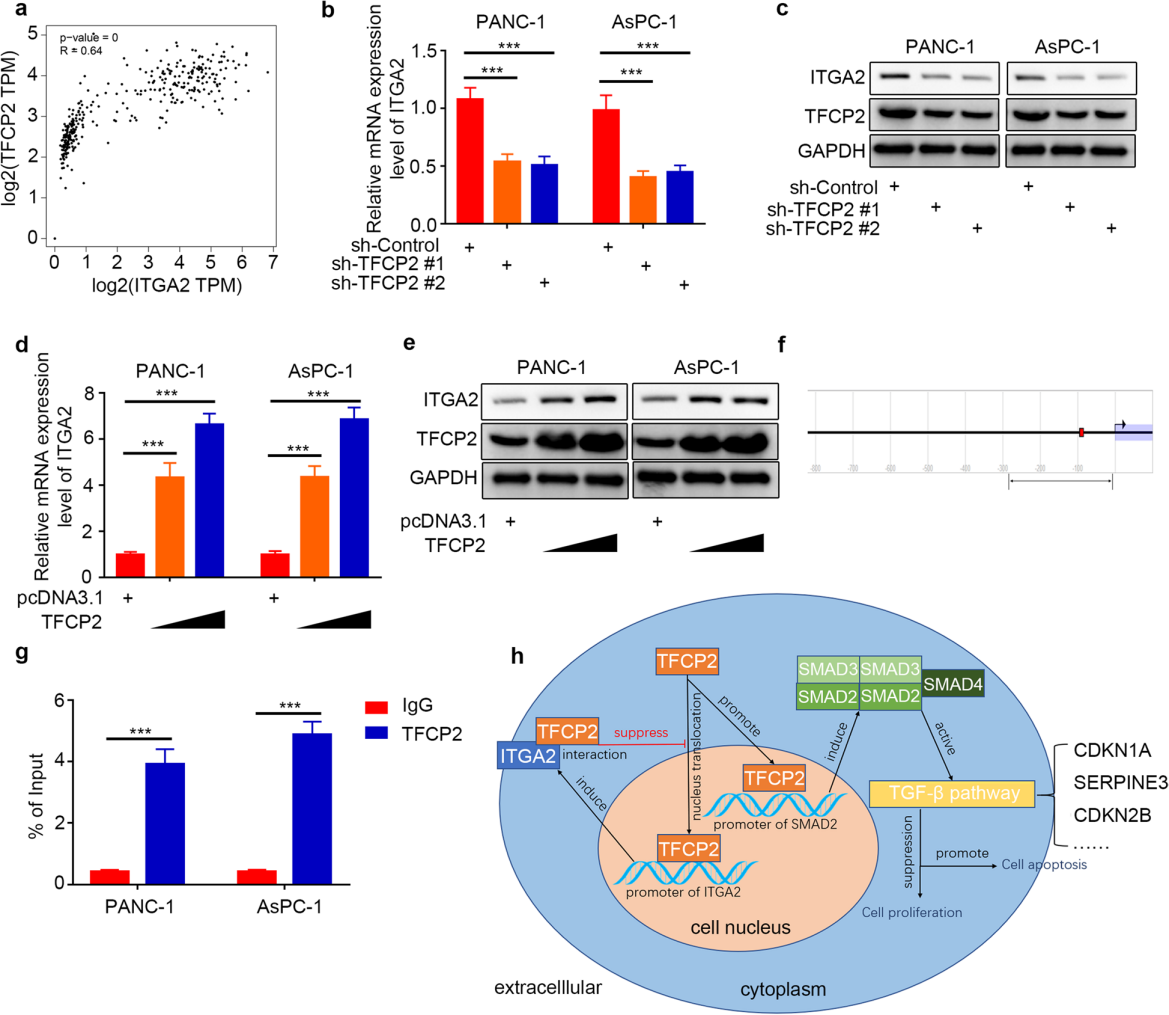
TFCP2 feedback induced the expression of ITGA2 as a transcription factor in pancreatic cancer cells. **a** GEPIA database was searched to determine the correlation between the mRNA expression of *TFCP2* and *ITGA2* in the PDAC samples. *P*-and R-values are indicated in the figure. **b** RT-PCR was used to determine the mRNA expression level of *ITGA2* in the PANC-1 and AsPC-1 cells infected with sh-Control or sh-TFCP2s. *GAPDH* served as an internal reference and repeated in triplicates. ns, not significant; ***P < 0.001. **c** Western blot analysis was used to determine the protein expression level of ITGA2 in the PANC-1 and AsPC-1 cells infected with sh-Control or sh-TFCP2s. GAPDH served as an internal reference. **d** RT-PCR was used to determine the mRNA expression level of *ITGA2* in the PANC-1 and AsPC-1 cells infected with pcDNA3.1 or TFCP2 plasmids. *GAPDH* served as an internal reference and repeated in triplicates. ns, not significant; ***P < 0.001. **e** Western blot analysis was used to determine the protein expression level of ITGA2 in the PANC-1 and AsPC-1 cells infected with pcDNA3.1 or TFCP2 plasmids. GAPDH served as an internal reference. **f** EPD database was searched to determine the potential binding sites of TFCP2 in the promoter region of *ITGA2* genes. **g** ChIP-qPCR of *TFCP2* in the PANC-1 and AsPC-1 cells. All the data are shown as the mean values ± SD from three independent replicates. ***P < 0.001. **h** Working model, showing that the ITGA2 inhibits the activation of TGF-β pathway in pancreatic cancer via TFCP2-SMAD2 axis, and TFCP2 feedback induced the expression of ITGA2 as a transcription factor

The previous results showed that ITGA2 could interact with TFCP2 at the protein level. However, searching the GEPIA database showed that the mRNA expression levels of TFCP2 positively correlated with the mRNA expression levels of ITGA2 in the pancreatic cancer cells (Fig. 8a). Therefore, TFCP2 might transcriptionally induce the ITGA2 expression by acting as a transcription factor in the pancreatic cancer cells. The RT-PCR and Western blot analyses showed that the *TFCP2* silencing significantly inhibited *ITGA2* expression transcriptionally (Fig. 8b and c), while the *TFCP2* overexpression significantly induced the *ITGA2* expression (Fig. 8d and e). In addition, searching the EPD database showed one potential binding site of TFCP2 in the promoter region of the *ITGA2* gene (Fig. 8f), which was verified using a ChIP-PCR assay (Fig. 8g). Therefore, these results indicated that the TFCP2 by acting as transcription factor feedback induced the ITGA2 expression in the pancreatic cancer cells.

## Discussion

Pancreatic cancer is one of the most invasive malignant tumors, with a 5-year survival rate of < 8% [16]. Radiotherapy, chemotherapy, and surgery are the main treatment methods, but the treatment outcome is poor. Therefore, targeted therapy for pancreatic cancer is the current mainstream trend to alleviate the disease and improve the living standards of patients.

Previous studies showed that ITGA2 is overexpressed in tumor cells and related to the poor prognosis of patients with cancer [6, 8–10, 20], especially pancreatic cancer [10]. However, the reason for the high expression level of ITGA2 in pancreatic cancer has not been clarified. Thus, we reviewed previous studies and unexpectedly found that ITGA2 overexpression was associated with the most common KRAS mutation in pancreatic cancer (Fig. 1). The carcinogenic mechanism of pancreatic cancer includes the mutation accumulation of KRAS [21], TP53, CDKN2A, and SMAD4 [22]. The abnormal movement of these molecules is the basis of the histological changes of pancreatic cancer at different stages of its development [22, 23]. Activated KRAS mutations were reported in > 90% of all pancreatic cancers [23].

Our previous research and recent local and foreign reports confirmed that the ITGA2 overexpression in pancreatic cancer is caused by the mutation and abnormal activation of KRAS. Previous studies reported that the carcinogenic mechanism of ITGA2 is rarely involved in pancreatic cancer, except that blocking ITGA2 can improve tumor immune response by reducing the phosphorylation level of STAT3 and inhibiting the PD-L1 expression [10]. Therefore, the purpose of this study was to examine other ways thereby ITGA2 regulates the progression of pancreatic cancer. For silenced ITGA2, RNA-seq was used to analyze the pathway enrichment of all genes. We found that the TGF-β signaling pathway was significantly activated when ITGA2 was silenced (Fig. 2). Furthermore, our results also indicated that ITGA2 silencing enhanced the anti-pancreatic cancer cell proliferation effect of TGF-β treatment, and the combined treatment might represent a novel therapeutic strategy for pancreatic cancer.

In the early stage of tumorigenesis, TGF-β expression can inhibit cell proliferation and participate in apoptosis. In the late stage of tumorigenesis, the TGF-β signaling pathway induces tumor invasion and metastasis by promoting angiogenesis, epithelial-mesenchymal transformation, and immune escape [24–26]. The transforming growth factor-β protein can regulate cellular function [27], which is essential for the homeostasis of epithelial cells, matrix compartments, and immune cells in the hepatopancreas and gastrointestinal system [28]. The transforming growth factor-β signaling pathway plays an important role in tumorigenesis by regulating cell proliferation, apoptosis, angiogenesis, immune surveillance, and metastasis [29–31]. The phosphorylation of the receptor-activated Smads (R-Smads) results in the formation of complexes with the common medium Smad (Co-Smad), which are introduced into the nucleus [32]. The TGF-β signaling pathway binds to SMAD4 through a complex composed of SMAD2 and SMAD3 [28]. The nuclear Smad oligomer binds to DNA and transcription factors to regulate the expression of the target gene [33–35]. In our present study, we conclude that ITGA2 affects the activation of the TGF-β pathway by regulating SMAD2, which is the key factor in the activation of the TGF-β pathway. Therefore, SMAD2 upregulation mediated the anti-tumor effect of ITGA2 silencing in pancreatic cancer, which represents treatment by inducing the SMAD2 expression as a novel therapeutic strategy for pancreatic cancer.

For tumor cells, transcription factor CP2 (TFCP2) expression can control the occurrence and regulate the development of tumor cells such as liver cancer [36]. In previous studies, TFCP2 was mostly considered a transcription factor that promotes the development and metastasis of cancer [36–38]. A few studies have reported that TFCP2 not only promotes but also inhibits cancers such as melanoma [19] and has some important physiological functions that have not yet been discovered [39]. TFCP2 expression participates in epithelial-mesenchymal transformation and enhances angiogenesis [19]. In our study, TFCP2, as a transcription factor of SMAD2, regulated the SMAD2 expression (Fig. 7), and ITGA2 expression influenced the nuclear translocation of TFCP2 by binding to TFCP2. Overexpression of ITGA2 hinders the entry of TFCP2 into the nucleus, resulting in increased and decreased TFCP2 expression levels outside and inside the nucleus, respectively (Fig. 7h, i). An interesting conclusion from this study is that TFCP2 may also be a transcription factor of ITGA2 (Fig. 8a–g), which forms a loop. When the TFCP2 expression level increases, ITGA2 binds to TFCP2 and inhibits its entry into the nucleus (Fig. 7h-j), resulting in decreases in SMAD2 and ITGA2 expression levels. The entry of TFCP2 into the nucleus increased, and the ITGA2 and SMAD2 expression levels recovered repeatedly, forming a dynamic balance.

## Conclusion

In conclusion, our findings show that overexpressed ITGA2 inhibits the SMAD2 expression by competitively binding to the transcription factor TFCP2 to block its entry into the nucleus, thus influencing the activation of the TGF-β signaling pathway, promoting tumor cell proliferation, and inhibiting tumor cell apoptosis in pancreatic cancer (Fig. 8h).

## Supplementary Information

**Additional file 1: Supplementary Table 1.** The shRNA sequences. **Supplementary Table 2.** The primer sequences for RT-qPCR. **Supplementary Table 3.** The primer sequences for ChIP-qPCR. **Supplementary Fig. 1.** Inhibition of TGF-β signaling could reverse the biological function conferred by ITGA2 depletion. (a and b) PANC-1 and AsPC-1 cells infected with sh-ITGA2 or sh-SMAD2 were harvested for colony formation assay (c) and CCK-8 cell proliferation assay (d). Each bar represents the mean ± SD of three independent experiments. ***P < 0.001. (c-e) Statistical analyses for protein level identified using Western blot analyses (Figs. 4d, h and 7e). Each bar represents the mean ± SD of three independent experiments. ***P < 0.001. **Supplementary Fig. 2.** Change in SMAD3 expression could affect the role of SMAD2 in the TGF-β signaling. (a-c) RT-PCR was used to determine the mRNA expression level of *CDKN2B* (a), *CDKN1A* (b) and *SERPINE3* (c) in the PANC-1 and AsPC-1 cells infected with sh-ITGA2 and/or sh-SMAD3. *GAPDH* served as an internal reference and repeated in triplicates. ns, not significant; ***P < 0.001. (d) Western blot analysis was used to determine the protein expression level of CDKN1A and CDKN2B in the PANC-1 and AsPC-1 cells infected with sh-ITGA2 and/or sh-SMAD3. GAPDH served as an internal reference.

## Data Availability

Please contact the corresponding author (rendianyun@hust.edu.cn) for data requests.

## Abbreviations

CCK-8: Cell Counting Kit-8
ChIP: Chromatin immunoprecipitation
qRT-PCR: Quantitative reverse; transcriptase polymerase chain reaction
GEO: Gene Expression Omnibus
TCGA: The Cancer Genome Atlas
GEPIA: Gene Expression Profiling Interactive Analysis
RNA-seq: RNA sequencing
siRNAs: Small interfering RNAs
GSEA: Gene Set Enrichment Analysis
SDS-PAGE: Sodium dodecyl sulfate polyacrylamide gel electrophoresis
BSA: Bovine serum albumin
PBS: Phosphate-buffered saline
DMEM: Dulbecco’s Modified Eagle’s Medium
ITGA2: Integrin alpha 2
TFCP2: Transcription factor CP2
Cleaved CASP3: Cleaved caspase3
FBS: Fetal bovine serum
KEGG: Kyoto Encyclopedia of Genes and Genomes
