# Supplementary material for: Overexpressed integrin alpha 2 inhibits the activation of the transforming growth factor β pathway in pancreatic cancer via the TFCP2-SMAD2 axis
Source: J Exp Clin Cancer Res. 2022 Feb 22;41:73. doi: 10.1186/s13046-022-02286-5 (PMC8862343; doi:10.1186/s13046-022-02286-5)
Supplement: Supplementary file 1 — Additional file 1: Supplementary Table 1. The shRNA sequences. Supplementary Table 2. The primer sequences for RT-qPCR. Supplementary Table 3. The primer sequences for ChIP-qPCR. Supplementary Fig. 1. Inhibition of TGF-β signaling could reverse the biological function conferred by ITGA2 depletion. (a and b) PANC-1 and AsPC-1 cells infected with sh-ITGA2 or sh-SMAD2 were harvested for colony formation assay (c) and CCK-8 cell proliferation assay (d). Each bar represents the mean ± SD of three independent experiments. ***P < 0.001. (c-e) Statistical analyses for protein level identified using Western blot analyses (Figs. 4d, h and 7e). Each bar represents the mean ± SD of three independent experiments. ***P < 0.001. Supplementary Fig. 2. Change in SMAD3 expression could affect the role of SMAD2 in the TGF-β signaling. (a-c) RT-PCR was used to determine the mRNA expression level of CDKN2B (a), CDKN1A (b) and SERPINE3 (c) in the PANC-1 and AsPC-1 cells infected with sh-ITGA2 and/or sh-SMAD3. GAPDH served as an internal reference and repeated in triplicates. ns, not significant; ***P < 0.001. (d) Western blot analysis was used to determine the protein expression level of CDKN1A and CDKN2B in the PANC-1 and AsPC-1 cells infected with sh-ITGA2 and/or sh-SMAD3. GAPDH served as an internal reference. [file 13046_2022_2286_MOESM1_ESM.docx]

**Overexpressed integrin alpha 2 inhibits the activation of the transforming growth factor β pathway in pancreatic cancer via the TFCP2-SMAD2 axis**

Hongkun Cai^1,2,#^, Feng Guo^1,2,#^, Shuang Wen ^3,#^, Xin Jin ^4,*^, Heshui Wu ^1, 2, *^, Dianyun Ren^1, 2, *^

^1^Department of Pancreatic Surgery, Union Hospital, Tongji Medical College, Huazhong University of Science and Technology, Wuhan 430022, China.

^2^Sino-German Laboratory of Personalized Medicine for Pancreatic Cancer, Union Hospital, Tongji Medical College, Huazhong University of Science and Technology, Wuhan 430022, China.

^3^Department of Emergency Medicine, Union Hospital, Tongji Medical College, Huazhong University of Science and Technology, Wuhan, Hubei, 430022, China.

^4^Department of Urology, The Second Xiangya Hospital, Central South University, Changsha, Hunan, 410011, China

^#^ These authors contributed equally to this work

***Corresponding author**: Dianyun Ren; Heshui Wu; Xin Jin

**E-mail address:**

Hongkun Cai ([hongkuncai@hust.edu.cn](mailto:hongkuncai@hust.edu.cn)); Feng Guo ([guofengunion@hust.edu.cn](mailto:guofengunion@hust.edu.cn));

Shuang Wen ([137428315@qq.com)](mailto:yansun@hust.edu.cn)); Xin Jin ([jinxinunion@hust.edu.cn)](mailto:jinxinunion@hust.edu.cn));

Heshui Wu ([heshuiwu@hust.edu.cn](mailto:heshuiwu@hust.edu.cn)); Dianyun Ren (ren[dianyun@hust.edu.cn](mailto:dianyun@hust.edu.cn));

**Supplementary Data**

**Supplementary table 1. The shRNA sequences.**

| **si-Control** | purchased from RIBOBIO |
| --- | --- |
| **si-ITGA2 #1** | CAGCCGCTACTTCCAATAT |
| **si-ITGA2 #2** | CAGGACTGGACATATCTGC |
| **sh-ITGA2 #1** | CCGGCCGGCCAGATAGTGCTATATACTCGAGTATATAGCACTATCTGGCCGGTTTTTG |
| **sh-ITGA2 #2** | CCGGATGGCAATATCACGGTTATTCCTCGAGGAATAACCGTGATATTGCCATTTTTTG |
| **sh-TFCP2 #1** | CCGGGCGCTGCTAGAAAGGGAAGATCTCGAGATCTTCCCTTTCTAGCAGCGCTTTTTG |
| **sh-TFCP2 #2** | CCGGGCTGCTAGAAAGGGAAGATTCCTCGAGGAATCTTCCCTTTCTAGCAGCTTTTTG |
| **sh-SMAD2 #1** | CCGGGCAGAACTATCTCCTACTACTCTCGAGAGTAGTAGGAGATAGTTCTGCTTTTTG |
| **sh-SMAD2 #2** | CCGGGCCAGTTACTTACTCAGAACCCTCGAGGGTTCTGAGTAAGTAACTGGCTTTTTG |

**Supplementary table 2.** **The primer sequences for RT-qPCR.**

| Gene | Forward primer (**5**′ - 3′) | Reverse primer (**5**′ - 3′) |
| --- | --- | --- |
| **GAPDH** | ATGACAATGAATACGGCTACAGCA | GCAGCGAACTTTATTGATGGTATT |
| **CDKN2B** | GGAATGCGCGAGGAGAACAA | CATCATCATGACCTGGATCGC |
| **CDKN1A** | CACCACTGGAGGGTGACTTC | ATCTGTCATGCTGGTCTGCC |
| **SERPINE3** | CCACCTCCGTGAAGGAATGAC | GGTAGTGTGGCATAAACAGCA |
| **SMAD2** | CGTCCATCTTGCCATTCACG | CTCAAGCTCATCTAATCGTCCTG |
| **SMAD3** | TGGACGCAGGTTCTCCAAAC | CCGGCTCGCAGTAGGTAAC |
| **SMAD4** | CCACCAAGTAATCGTGCATCG | TGGTAGCATTAGACTCAGATGGG |
| **TFCP2** | TCTGGCCGACGAAGTGATTG | ATCAGGAGGCAAACTCGACTC |
| **ITGA2** | GGGAATCAGTATTACACAACGGG | CCACAACATCTATGAGGGAAGGG |

**Supplementary table 3.** **The primer sequences for ChIP-qPCR.**

| Gene | Forward primer (**5**′ - 3′) | Reverse primer (**5**′ - 3′) |
| --- | --- | --- |
| **SMAD2** | AAGACCCTTCGGGACATGGA | CGCGCATTAAGACGATTCCC |
| **ITGA2** | CGGATATGCCCACCCGTC | CGGTGAGAGCAGGGAAAAGT |

**Supplementary figure 1**


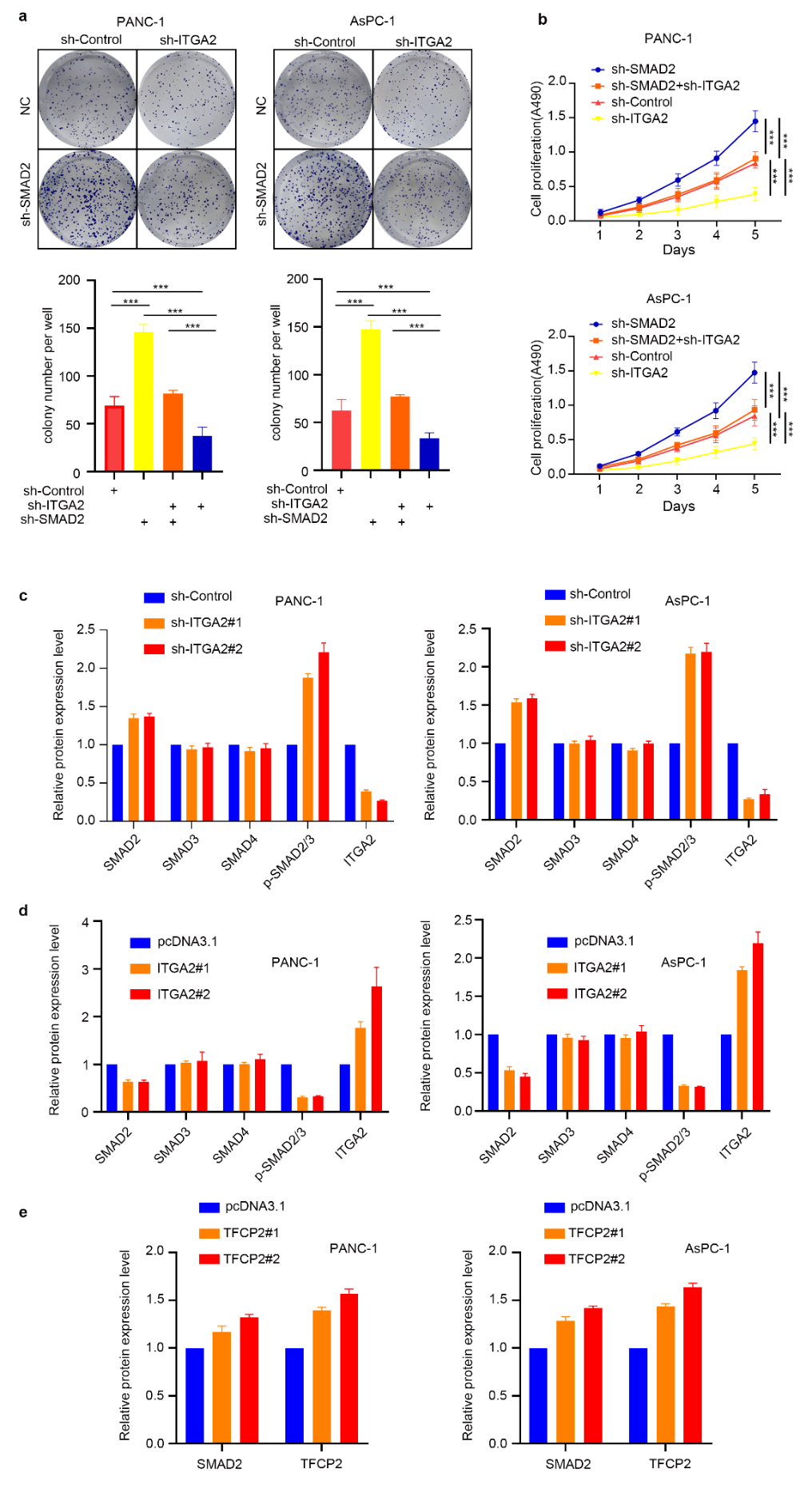


**Supplementary figure 1. Inhibition of TGF-β signaling could reverse the biological function conferred by ITGA2 depletion.**

**(a and b)** PANC-1 and AsPC-1 cells infected with sh-ITGA2 or sh-SMAD2 were harvested for colony formation assay (c) and CCK-8 cell proliferation assay (d). Each bar represents the mean ± SD of three independent experiments. ****P* <0.001. **(c-e)** Statistical analyses for protein level identified using Western blot analyses (Figs. 4d, 4h and 7e). Each bar represents the mean ± SD of three independent experiments. ****P* <0.001.

**Supplementary figure 2**


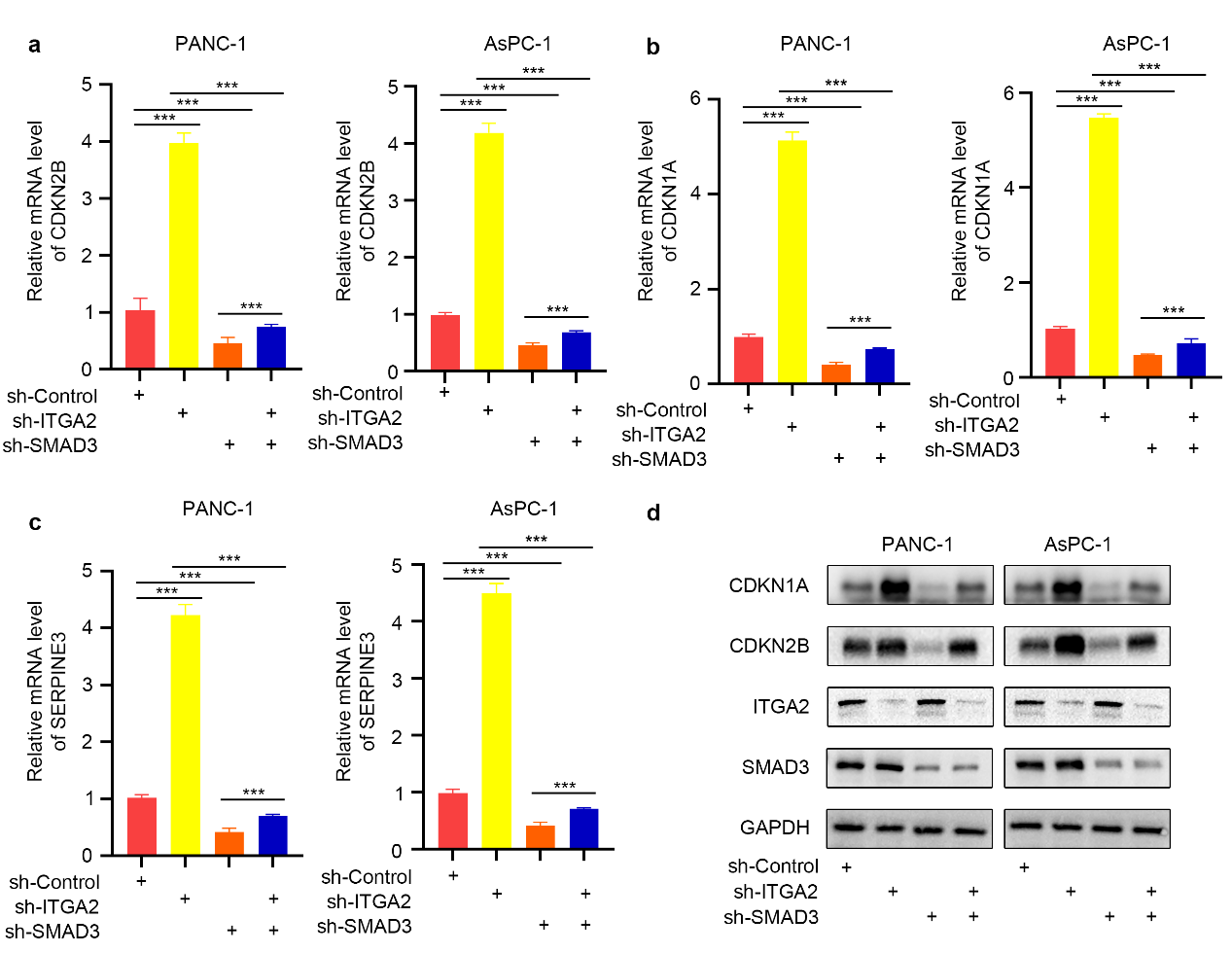


**Supplementary figure 2. Change in SMAD3 expression could affect the role of SMAD2 in the TGF-β signaling. (a-c)** RT-PCR was used to determine the mRNA expression level of *CDKN2B* (a), *CDKN1A* (b) and *SERPINE3* (c) in the PANC-1 and AsPC-1 cells infected with sh-ITGA2 and/or sh-SMAD3. *GAPDH* served as an internal reference and repeated in triplicates. ns, not significant; ****P* <0.001. **(d)** Western blot analysis was used to determine the protein expression level of CDKN1A and CDKN2B in the PANC-1 and AsPC-1 cells infected with sh-ITGA2 and/or sh-SMAD3. GAPDH served as an internal reference.
